# Prognosis of Periapical Lesions Treated by Activated Disinfection (PUI, Laser) Without the Use of Systemic Antibiotics: Systematic Review and Meta-Analysis

**DOI:** 10.3390/jcm15145397

**Published:** 2026-07-09

**Authors:** Paula Fernández-Moreno, Victoria Areal-Quecuty, Carlos Segura-Raya, Juan J. Saúco-Márquez, Benito Sánchez-Domínguez, Milagros Martín-Jiménez, Juan J. Segura-Egea, María León-López

**Affiliations:** 1 Endodontic Section, Department of Stomatology, School of Dentistry, University of Sevilla, C/Avicena s/n, 41009 Sevilla, Spain; 3bpaulafernandez@gmail.com (P.F.-M.); vareal@us.es (V.A.-Q.); seraca01@gmail.com (C.S.-R.); jjsauco@us.es (J.J.S.-M.); mmartin33@us.es (M.M.-J.); 2 Andalusian Health Service, Distrito Sanitario Sevilla, 41013 Sevilla, Spain; beni2506@yahoo.es; 3 Faculty of Health Sciences and Sports, University Center San Isidoro affiliated with Pablo de Olavide University, 41092 Sevilla, Spain

**Keywords:** apical periodontitis, root canal therapy, periapical lesions, activated irrigation, passive ultrasonic irrigation, laser-activated irrigation, PUI, laser disinfection, root canal disinfection

## Abstract

**Background/Objectives:** Activated irrigation techniques improve intracanal disinfection, but their impact on the clinical and radiographic healing of apical periodontitis remains unclear. This systematic review and meta-analysis evaluated the efficacy of passive ultrasonic irrigation (PUI) and laser-assisted irrigation (LAI), without adjunctive systemic antibiotics, on periapical healing compared with conventional irrigation in adult patients. **Methods:** Following PRISMA guidelines and prospective registration in PROSPERO (CRD420261413401), PubMed, Scopus, Embase, and Web of Science were searched up to February 2026. Randomized controlled trials (RCTs) involving adults with apical periodontitis comparing PUI or LAI against conventional syringe irrigation—with a minimum of 6 months follow-up—were included. Risk of bias was assessed using RoB 2, evidence certainty via GRADE, and a random-effects meta-analysis calculated pooled odds ratios (ORs) and 95% confidence intervals (CIs). **Results:** Of 1115 records identified, five randomized controlled trials involving 451 teeth fulfilled the eligibility criteria and were included in the qualitative synthesis and quantitative meta-analysis. Activated irrigation significantly increased periapical healing probability compared with conventional irrigation (OR = 2.25; 95% CI: 1.29–3.93; *p* = 0.004), with no statistical heterogeneity (I^2^ = 0%). Subgroup analyses showed significant benefits for both PUI (OR = 1.95; 95% CI: 1.08–3.54) and LAI (OR = 6.77; 95% CI: 1.63–28.12). The overall certainty of evidence was moderate due to risk of bias concerns. **Conclusions:** Activated irrigation techniques (PUI and LAI) were significantly associated with improved clinical and radiographic healing of apical periodontitis compared with conventional irrigation alone. Enhanced intracanal disinfection contributes to a more predictable resolution of periapical lesions without adjunctive systemic antibiotics. Further high-quality RCTs with standardized protocols and long-term CBCT-based follow-up are required to confirm these findings.

## 1. Introduction

Apical periodontitis (AP) is an infectious inflammatory disease of the periradicular tissues caused by microbial colonization of the root canal system, usually following pulp necrosis [1]. Intradental biofilms and their by-products trigger host immune responses that result in periapical bone destruction, frequently detected radiographically as an apical radiolucency [2,3].

The primary objective of root canal treatment is to eliminate microorganisms and necrotic tissue from the root canal system. However, the complex root canal anatomy, including isthmuses, fins, and apical ramifications, limits the effectiveness of mechanical instrumentation alone and may allow bacterial persistence in inaccessible areas [4,5]. Therefore, chemical irrigation plays a crucial role in endodontic disinfection. Although sodium hypochlorite (NaOCl) and EDTA remain the gold-standard irrigants, conventional syringe-and-needle irrigation may not achieve adequate irrigant penetration in the apical region because of physical limitations, including the vapor lock phenomenon [5,6].

To overcome these limitations, several irrigant activation techniques have been developed. Among them, passive ultrasonic irrigation (PUI) enhances irrigant dynamics through acoustic streaming and cavitation generated by a non-cutting ultrasonically activated tip, facilitating the removal of debris and biofilm from anatomically complex areas of the root canal system [7,8,9]. More recently, several laser-assisted irrigation systems have gained relevance in endodontics. Er technologies, including photon-induced photoacoustic streaming (PIPS) and shock-wave enhanced emission photoacoustic streaming (SWEEPS), generate photoacoustic effects through strong absorption in irrigant solutions. Other laser systems, such as Er,Cr and Nd lasers, have also been investigated for endodontic disinfection, although their physical properties and mechanisms of action differ from those of Er lasers. These technologies utilize photoacoustic shock waves to intensely agitate the irrigant, allowing minimally invasive approaches without requiring deep fiber insertion [10,11,12].

Although in vitro and ex vivo studies have consistently shown that passive ultrasonic irrigation (PUI) and laser-assisted irrigation (LAI) improve irrigant penetration, biofilm disruption, and debris removal compared with conventional syringe irrigation, the clinical significance of these advantages remains uncertain [9,13]. Since the ultimate goal of endodontic treatment is the clinical and radiographic healing of apical periodontitis, rather than microbial reduction alone, it is essential to determine whether the enhanced disinfection achieved by activated irrigation translates into improved healing outcomes. However, clinical studies have reported inconsistent results, and previous systematic reviews have concluded that the available evidence is insufficient to establish a clear benefit of activated irrigation on periapical healing [5,14].

Furthermore, the use of systemic antibiotics in some treatment protocols may act as a confounding factor, making it difficult to isolate the specific contribution of intracanal disinfection to healing outcomes. Because current international guidelines recommend systemic antibiotics only in cases of systemic involvement, spreading infection, or medically compromised patients [2], evaluating activated irrigation techniques in the absence of adjunctive antibiotic therapy is both clinically relevant and methodologically justified [15]. Moreover, previous reviews have mainly focused on surrogate outcomes such as bacterial reduction, smear layer removal, or postoperative pain, whereas evidence specifically addressing the healing of periapical lesions remains limited [2,14,16].

Therefore, this systematic review and meta-analysis aimed to evaluate whether activated root canal disinfection using PUI and/or laser systems, without systemic antibiotic therapy, improves the medium- and long-term clinical and radiographic healing of periapical lesions compared with conventional irrigation techniques. We hypothesized that activated irrigation techniques, including passive ultrasonic irrigation and laser-assisted irrigation, would be associated with higher clinical and radiographic healing rates of apical periodontitis than conventional syringe-and-needle irrigation when used without adjunctive systemic antibiotic therapy.

## 2. Materials and Methods

The methodology of this systematic review was conducted and reported according to the Preferred Reporting Items for Systematic Reviews and Meta-Analyses (PRISMA) 2020 statement [17]. The completed PRISMA 2020 Checklist is available in the Supplementary Materials (Supplementary Table S1). The protocol was prospectively registered in PROSPERO prior to study initiation (CRD420261413401).

### 2.1. Research Question and Study Design

To ensure a structured and reproducible approach, the research question was formulated according to the PICOT framework (Population, Intervention, Comparison, Outcome, and Time):

- Population (P): Adult patients (≥18 years old) with permanent teeth presenting radiographically diagnosed periapical lesions and requiring endodontic treatment.
- Intervention (I): Intracanal irrigant activation using passive ultrasonic irrigation (PUI) and/or laser systems, without the adjunctive use of systemic antibiotics.
- Comparison (C): Conventional, non-activated syringe and needle irrigation using the same irrigant solutions, without the adjunctive use of systemic antibiotics.
- Outcome (O): Clinical healing (absence of signs and symptoms) and/or radiographic healing (reduction or disappearance of the periapical lesion).
- Time (T): Medium-term (≥6 months) and long-term (≥12 months) post-treatment follow-up.

Accordingly, the following clinical question was addressed: In adult patients with radiographically diagnosed periapical lesions requiring root canal treatment, activated irrigation using PUI or laser-assisted techniques, in the absence of systemic antibiotic therapy, improve medium- and long-term clinical and radiographic healing compared with conventional irrigation?

### 2.2. Eligibility Criteria

Studies were selected for inclusion based on specific criteria derived from the PICOT framework:

#### 2.2.1. Inclusion Criteria

Studies were considered eligible if they met all of the following criteria:

Study design: Randomized controlled trials (RCTs).

Population: Adult patients (≥18 years old) with permanent teeth presenting radiographically diagnosed apical periodontitis or periapical lesions and requiring primary or secondary root canal treatment.

Intervention: Intracanal irrigant activation using passive ultrasonic irrigation (PUI) and/or laser-assisted irrigation (LAI), performed without adjunctive systemic antibiotic therapy.

Comparison: Conventional syringe-and-needle irrigation using the same irrigating solution and concentration as the intervention group, without adjunctive systemic antibiotic therapy.

Outcomes: Studies reporting clinical healing (absence of pain, swelling, sinus tract, or other signs and symptoms) and/or radiographic healing (reduction or complete resolution of the periapical radiolucency).

Follow-up period: Minimum postoperative follow-up of 6 months, with preference given to studies reporting medium-term (≥6 months) and long-term (≥12 months) healing outcomes.

Publication characteristics: Full-text articles published in peer-reviewed journals, without restrictions regarding publication year or language.

#### 2.2.2. Exclusion Criteria

Studies were excluded if they met any of the following criteria:

Non-clinical studies, including in vitro, ex vivo, animal, finite-element, or laboratory-based investigations.

Non-randomized study designs, including observational studies, cohort studies, case–control studies, case series, case reports, narrative reviews, systematic reviews, editorials, letters to the editor, conference abstracts, and expert opinions.

Ineligible population, including patients younger than 18 years of age, primary teeth, permanent teeth without radiographic evidence of apical periodontitis, or studies involving surgically treated lesions.

Use of systemic antibiotics, either routinely administered as part of the treatment protocol or when the study failed to clearly report whether systemic antibiotic therapy had been prescribed.

Ineligible interventions, including studies evaluating activation techniques other than PUI or laser-assisted irrigation (e.g., sonic activation, negative-pressure irrigation, continuous ultrasonic irrigation, or other adjunctive disinfection methods).

Non-comparable irrigation protocols, defined as studies in which the intervention and control groups differed in the type, concentration, or composition of the irrigating solution, thereby preventing the isolated evaluation of the activation technique.

Insufficient follow-up, defined as a postoperative follow-up period shorter than 6 months.

Inadequate outcome reporting, including studies that did not provide clear clinical and/or radiographic healing outcomes or lacked sufficient data for extraction and quantitative synthesis.

### 2.3. Literature Search Strategy and Study Selection

A comprehensive electronic search was systematically conducted across the PubMed/MEDLINE, Scopus, Embase, and Web of Science (WOS) databases for relevant articles published up to 3 February 2026, without language or publication year restrictions.

The search strategies were developed using a combination of Medical Subject Headings (MeSH), Emtree terms, and free-text keywords linked with Boolean operators. The search syntax was adapted to the specific requirements of each database. The complete search strategies for all databases are showed in Table 1.

In addition, the reference lists of all included studies and relevant review articles were manually screened to identify potentially eligible studies not captured through the electronic search.

All retrieved records were imported into Zotero 8.0.3 (Corporation for Digital Scholarship, Vienna, VA, USA), where duplicate records were identified and removed, followed by manual verification. Two reviewers (INICIALES) independently screened titles and abstracts according to the predefined eligibility criteria. Full-text articles were subsequently assessed for all potentially relevant studies. Any disagreements were resolved by discussion; when consensus could not be reached, a third reviewer made the final decision.

### 2.4. Data Extraction and Statistical Analysis

Data extraction was performed independently by two reviewers (P. F-M and M. L-L) using a standardized data collection form. The following information was extracted from each eligible study: author, country, year of publication, study design, sample size, population characteristics, intervention protocol (PUI and/or laser-assisted irrigation), control protocol (conventional irrigation), clinical and radiographic outcomes, and main findings. The primary outcome was treatment success, defined according to the criteria reported by each study as clinical and/or radiographic healing of the periapical lesion at the longest available follow-up. Additional variables included tooth type, pulpal and periapical diagnosis, irrigant type and concentration, activation protocol, and follow-up duration.

A meta-analysis was performed to compare the probability of healing success between activated irrigation and conventional irrigation. Statistical analyses were conducted using OpenMeta [Analyst] software (version 10.10) (Brown University, Providence, RI, USA) [18]. Pooled effect estimates were calculated as odds ratios (ORs) with corresponding 95% confidence intervals (CIs) using a random-effects model according to the DerSimonian and Laird method. Odds ratios were selected as the effect measure because the primary outcome (healing versus non-healing) was dichotomous and ORs are commonly used in meta-analyses of randomized controlled trials evaluating treatment effects. For multi-arm studies, each eligible intervention group (PUI and/or laser-assisted irrigation) was compared separately with the corresponding conventional irrigation control group. A random-effects model was selected a priori because clinical heterogeneity was expected due to differences in patient populations, activation protocols, irrigant concentrations, and follow-up periods.

Statistical heterogeneity among studies was assessed using the I^2^ statistic. Values of 25–50%, 50–75%, and >75% were considered indicative of low, moderate, and substantial heterogeneity, respectively. Statistical significance was established at *p* < 0.05. Publication bias was not formally assessed because fewer than ten studies were included in the meta-analysis, in accordance with current methodological recommendations.

### 2.5. Risk of Bias Assessment

The methodological quality of the included randomized controlled trials was assessed independently by two reviewers using the Cochrane Risk of Bias 2 (RoB 2) tool [19]. Disagreements were resolved by discussion and, when necessary, by consultation with a third reviewer. The following domains were evaluated: bias arising from the randomization process, bias due to deviations from intended interventions, bias due to missing outcome data, bias in outcome measurement, and bias in the selection of the reported results. Each study was classified as having a low risk of bias, some concerns, or a high risk of bias according to the RoB 2 guidance.

Publication bias was not formally assessed because fewer than ten studies were included in the meta-analysis.

### 2.6. Quality Assessment and Certainty of Evidence

The overall certainty of the evidence for the primary outcomes was assessed using the Grading of Recommendations Assessment, Development and Evaluation (GRADE) approach [20]. The certainty of evidence was rated as high, moderate, low, or very low based on the risk of bias, inconsistency, indirectness, imprecision, and publication bias. A high certainty rating indicates strong confidence that the true effect is close to the estimated effect, whereas low or very low certainty indicates limited confidence in the effect estimate [21]. The certainty of evidence assessment was conducted independently by two reviewers using the GRADE approach. Any disagreements were resolved through consensus or adjudication by a third reviewer.

## 3. Results

### 3.1. Study Selection and Flow Diagram

**Figure 1:**
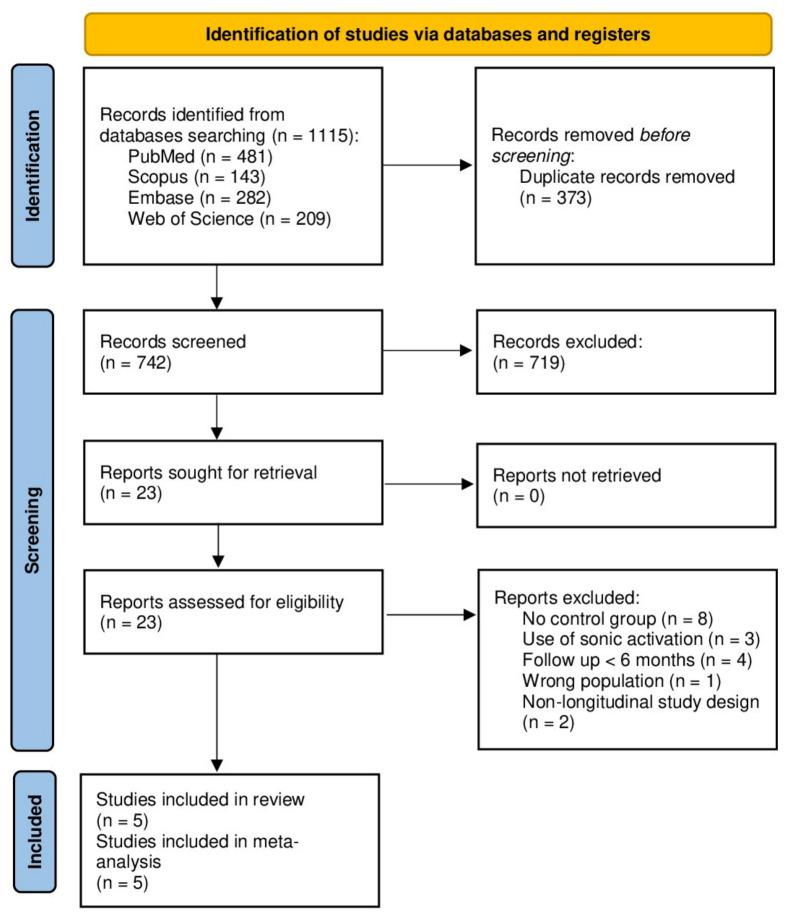
Flowchart of the search strategy following the PRISMA 2020 guidelines for systematic reviews and meta-analyses [17].

The entire screening and selection process is visually summarized in the PRISMA flow diagram (Figure 1) [17].

The initial database search yielded 1115 records. After removal of duplicate citations (*n* = 373), 742 records were screened by title and abstract, resulting in the exclusion of 719 studies. A total of 23 reports were considered potentially relevant and were sought for retrieval. Of these, all 23 full-text articles were successfully retrieved and moved forward for formal eligibility assessment.

Nineteen studies were excluded after full-text evaluation for the following reasons: absence of an appropriate conventional irrigation control group (*n* = 8), use of sonic activation instead of PUI or laser activation (*n* = 3), follow-up shorter than 6 months (*n* = 4), non-longitudinal study design (*n* = 2), and ineligible patient population (*n* = 1) (Table 2). Ultimately, five randomized controlled trials met all eligibility criteria and were included in both the qualitative synthesis and quantitative meta-analysis [22,23,24,25,26].

### 3.2. Characteristics of the Included Studies

The characteristics of the five studies selected and included for systematic review and meta-analysis are shown in Table 3.

The five included studies were randomized controlled trials conducted in India, China, Turkey, and Portugal [22,23,24,25,26]. All studies compared activated irrigation techniques—passive ultrasonic irrigation (PUI), laser-assisted irrigation (LAI), or both—with conventional syringe-and-needle irrigation. Sodium hypochlorite (NaOCl) was used as the primary irrigant in all studies, with concentrations ranging from 2.5% to 5.25%. Differences among studies mainly concerned the activation protocol, follow-up duration (6–19 months), and radiographic assessment methods, which included periapical radiography and/or cone-beam computed tomography (CBCT). Two studies evaluated PUI exclusively [23,24], one evaluated laser activation alone [26], and two included both activation modalities within a multi-arm design [22,25].

Although all studies evaluated clinical and radiographic healing, the criteria used to define treatment success were not completely uniform. Some studies considered complete radiographic healing as the primary endpoint, whereas others also classified substantial lesion reduction in the absence of clinical signs and symptoms as a successful outcome.

Overall, all studies reported favorable healing outcomes following root canal treatment, regardless of the irrigation protocol employed. Verma et al. [22] reported significantly higher success rates for both the PUI (100%) and LAI (100%) groups compared with the conventional irrigation group (78.9%; *p* = 0.014) after 12 months of follow-up, with no significant differences between the two activation techniques. Tang et al. [23] observed higher healing rates in the activated groups than in the conventional irrigation group at both 6 and 12 months; however, these differences were not statistically significant (*p* = 0.375 and *p* = 0.078, respectively). Similarly, Liang et al. [24] reported high healing rates in both the PUI (95.1%) and control (88.4%) groups, without significant intergroup differences (*p* = 0.470). Using CBCT volumetric analysis, Doğan et al. [25] demonstrated a significant reduction in lesion size over time in all treatment groups (*p* < 0.05), although no significant differences were detected among the activation protocols and conventional irrigation (*p* = 0.414). Likewise, Martins et al. [26] found that laser-assisted irrigation achieved clinical and radiographic healing outcomes comparable to those obtained with conventional irrigation after 12 months of follow-up.

Taken together, the included studies consistently demonstrated that root canal treatment was associated with favorable periapical healing outcomes. Although activated irrigation techniques generally showed higher success rates than conventional irrigation, statistically significant superiority was demonstrated in only one study [22].

Important methodological differences were identified among the included studies. Radiographic healing was assessed using either conventional periapical radiography or CBCT, the latter providing a more sensitive detection of periapical changes. Furthermore, treatment success was not uniformly defined across studies, with some authors using complete radiographic healing as the primary endpoint, whereas others considered substantial lesion reduction or combined clinical and radiographic criteria. Follow-up periods also varied between 6 and 12 months. These differences should be considered when interpreting the pooled estimates and may contribute to clinical heterogeneity despite the absence of statistical heterogeneity.

The outcome measures reported by the included studies were not entirely uniform. Therefore, the percentages presented in Table 3 reflect study-specific outcome categories, whereas Table 4 summarizes the treatment success rates used for the meta-analysis.

### 3.3. Quantitative Synthesis and Meta-Analysis

A quantitative synthesis was performed using data from the five included randomized controlled trials [22,23,24,25,26]. The meta-analysis assessed the effect of activated irrigation techniques on the probability of periapical healing compared with conventional irrigation. The characteristics of the studies included in the meta-analysis, together with the number of successful outcomes, healing rates, and study-specific Odds Ratios (ORs), are presented in Table 4. The table provides the data used to compare activated irrigation techniques (PUI and/or laser-assisted irrigation) with conventional irrigation.

#### 3.3.1. Primary Outcome: Clinical and Radiographic Healing

**Figure 2:**
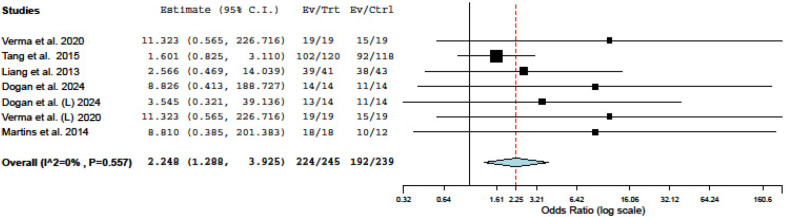
Forest plot of the random-effects meta-analysis for clinical and radiographic healing rates of periapical lesions treated with activated irrigation modalities versus conventional syringe irrigation. Individual included studies are cited as follows: Liang et al. 2013 [24], Martins et al. 2014 [26], Tang et al. 2015 [23], Verma et al. 2020 [22], Dogan et al. 2024 [25]. The black squares represent the weight and effect size of individual trials, and the horizontal lines represent their 95% confidence intervals. The vertical solid black line indicates the line of no effect (relative risk/odds ratio = 1). The vertical red line denotes the overall pooled effect size, passing through the center of the diamond that represents the combined statistical outcome.

The overall pooled effect is shown in the forest plot presented in Figure 2. The primary outcome was the overall treatment success rate, defined as clinical and/or radiographic healing of periapical lesions. This outcome was used to compare activated irrigation techniques (PUI and/or laser-assisted irrigation) with conventional syringe-and-needle irrigation. A subgroup analysis was also conducted according to the activation technique employed.

As shown in Figure 2, activated irrigation was associated with a significantly higher probability of treatment success than conventional irrigation (OR = 2.25; 95% CI: 1.29–3.93; *p* = 0.004). These findings indicate an overall benefit of activated irrigation in the management of periapical lesions.

No statistical heterogeneity was detected among the included studies (I^2^ = 0%; Cochran’s Q-test, *p* = 0.557), suggesting a high degree of consistency in the direction of the treatment effect across studies. The pooled effect estimate remained statistically significant, supporting the association between activated irrigation and improved healing outcomes.

#### 3.3.2. Subgroup Analysis

**Figure 3:**
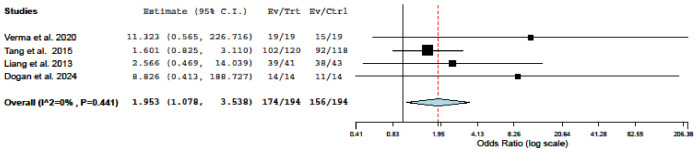
Forest plot of the subgroup analysis comparing passive ultrasonic irrigation (PUI) versus conventional syringe irrigation for the healing of periapical lesions. Individual included studies for this subgroup are cited as follows: Liang et al. 2013 [24], Tang et al. 2015 [23], Verma et al. 2020 [22], and Dogan et al. 2024 [25]. The black squares represent the weight and effect size of individual trials, and the horizontal lines represent their 95% confidence intervals. The vertical solid black line indicates the line of no effect. The vertical red line denotes the overall pooled effect size, passing through the center of the diamond that represents the combined statistical outcome for this subgroup.

**Figure 4:**
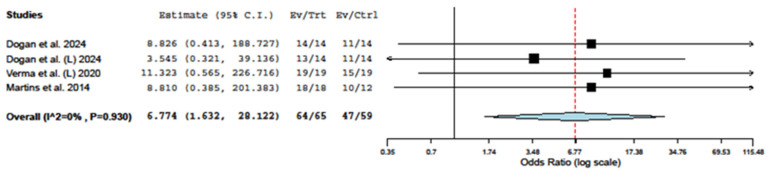
Forest plot of the subgroup analysis comparing laser-activated irrigation (LAI) versus conventional syringe irrigation for the healing of periapical lesions. Individual included studies for this subgroup are cited as follows: Martins et al. 2014 [26], Verma et al. 2020 [22], and Dogan et al. 2024 [25]. The black squares represent the weight and effect size of individual trials, and the horizontal lines represent their 95% confidence intervals. The vertical solid black line indicates the line of no effect. The vertical red line denotes the overall pooled effect size, passing through the center of the diamond that represents the combined statistical outcome for this subgroup.

To evaluate whether the treatment effect differed according to the activation method used, separate subgroup analyses were conducted for passive ultrasonic irrigation (PUI) and laser-assisted irrigation (LAI). The corresponding forest plots are shown in Figure 3 and Figure 4, respectively.

The subgroup analysis evaluating passive ultrasonic irrigation (PUI) demonstrated a significantly higher probability of periapical healing compared with conventional irrigation (OR = 1.95; 95% CI: 1.08–3.54). As shown in Figure 3, all included studies showed a treatment effect favoring PUI, although the magnitude of the effect varied among studies. The largest effect estimates were observed in the studies by Verma et al. [22] and Doğan et al. [25], whereas Tang et al. [23] and Liang et al. [24] reported more modest differences between groups.

No statistical heterogeneity was detected among the included studies (I^2^ = 0%; *p* = 0.441), indicating a high degree of consistency in the direction of the treatment effect. Overall, these findings suggest that PUI is associated with significantly improved healing outcomes compared with conventional syringe-and-needle irrigation.

The subgroup analysis evaluating laser-assisted irrigation (LAI) demonstrated a significantly higher probability of periapical healing compared with conventional irrigation (OR = 6.77; 95% CI: 1.63–28.12). As shown in Figure 4, all included studies favored LAI over conventional syringe-and-needle irrigation, although considerable variation was observed in the magnitude of the effect estimates. The largest treatment effects were reported by Verma et al. [22] and Martins et al. [26], whereas Doğan et al. [25] showed a more modest, although still favorable, effect.

No statistical heterogeneity was detected among the included studies (I^2^ = 0%; *p* = 0.930), indicating a high degree of consistency in the direction of the treatment effect. Overall, these findings suggest that LAI is associated with significantly improved healing outcomes compared with conventional irrigation.

### 3.4. Results

**Figure 5:**
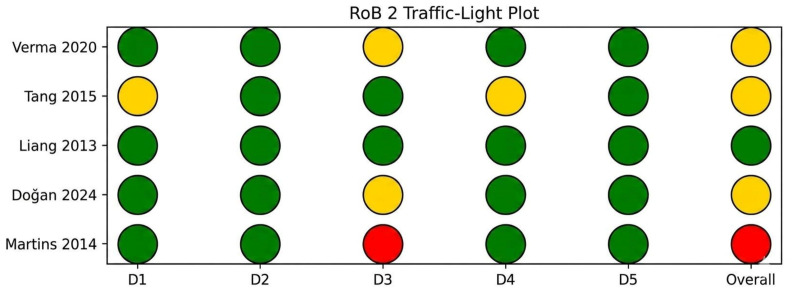
Traffic-light plot presenting the risk of bias assessment for each included randomized controlled trial across individual domains using the Cochrane RoB 2 tool. Individual evaluated studies are cited as follows: Liang et al. 2013 [24], Martins et al. 2014 [26], Tang et al. 2015 [23], Verma et al. 2020 [22], and Dogan et al. 2024 [25]. The colors within the plot represent the risk level for each specific domain: green circles indicate a low risk of bias, yellow circles represent some concerns, and red circles indicate a high risk of bias. (D1: Bias arising from the randomization process; D2: Bias due to deviations from intended interventions; D3: Bias due to missing outcome data; D4: Bias in outcome measurement; D5: Bias in the selection of the reported result).

The risk of bias assessment of the included randomized controlled trials is summarized in Figure 5. Among the five studies evaluated, one study was judged to have a low overall risk of bias [24], three studies were classified as presenting some concerns [22,23,25], and one study was considered at high risk of bias [26].

Regarding the individual domains, the risk of bias arising from deviations from intended interventions (D2) and the selection of the reported results (D5) was rated as low across all included studies. The randomization process (D1) was generally adequate, although Tang et al. [23] raised some concerns due to insufficient information regarding allocation procedures. Missing outcome data (D3) represented the most frequent source of potential bias, leading to some concerns in Verma et al. [22] and Doğan et al. [25], and a high risk of bias in Martins et al. [26]. Bias in outcome measurement (D4) was considered low in most studies, with some concerns identified only in Tang et al. [23].

Overall, the included studies demonstrated predominantly low risk of bias or some concerns, with only one study classified as having a high overall risk of bias.

### 3.5. GRADE Assessment of Quality of Evidence

The certainty of the evidence for the primary outcome was assessed using the GRADE approach and is summarized in Table 5. Overall, the certainty of the evidence was rated as moderate (⊕⊕⊕◯). Although all included studies were randomized controlled trials, the certainty was downgraded by one level due to concerns regarding risk of bias. Specifically, three studies were judged as having some concerns and one study was classified as high risk of bias according to the RoB 2 assessment.

No serious inconsistency was identified among the included studies, as reflected by the absence of statistical heterogeneity in the meta-analysis (I^2^ = 0%; *p* = 0.557). Similarly, no serious concerns were identified regarding indirectness, as all included studies directly addressed the review question. Although some individual studies reported relatively wide confidence intervals, the pooled effect estimate remained statistically significant (OR = 2.25; 95% CI: 1.29–3.93) and did not cross the line of no effect; therefore, imprecision was not considered sufficiently serious to warrant downgrading the certainty of evidence. Consequently, the available evidence provides moderate certainty that activated irrigation techniques are associated with improved healing outcomes compared with conventional irrigation.

High certainty: There is high confidence that the true effect lies close to that of the estimate.
Moderate certainty: There is moderate confidence in the effect estimate: the true effect is likely to be close to the estimate of the effect, but there is a possibility that it is substantially different.
Low certainty: Confidence in the effect estimate is limited; the true effect may be substantially different from the estimate.
Very low certainty: There is very little confidence in the effect estimate; the true effect is likely to be substantially different from the estimate.

## 4. Discussion

The objective of the present study was to evaluate, through a systematic review and meta-analysis, whether activated disinfection of the root canal system using passive ultrasonic irrigation (PUI) or laser activation, in the absence of systemic antibiotics, was associated with a higher probability of healing of periapical lesions compared to conventional irrigation.

### 4.1. Main Findings

The results of the present systematic review and meta-analysis demonstrated that activated irrigation techniques, including passive ultrasonic irrigation (PUI) and laser-assisted irrigation (LAI), significantly increased the probability of clinical and radiographic healing of apical periodontitis compared with conventional syringe-and-needle irrigation. The pooled analysis revealed that teeth treated with activated disinfection were more than twice as likely to achieve successful healing than those treated with conventional irrigation alone (OR = 2.25; 95% CI: 1.29–3.93). Although all treatment protocols resulted in favorable healing outcomes, the findings suggest that enhanced intracanal disinfection may contribute to a more predictable resolution of periapical lesions. Moreover, the absence of statistical heterogeneity among the included studies (I^2^ = 0%) strengthens the consistency of the observed effect despite differences in activation protocols, imaging methods, and follow-up periods.

### 4.2. Biological Rationale for Improved Periapical Healing with Activated Irrigation

The favorable effect of activated irrigation on periapical healing is biologically plausible and can be explained by its enhanced ability to disrupt and eliminate intracanal biofilms. Persistent microbial infection within the root canal system is widely recognized as the primary etiological factor responsible for the development and maintenance of apical periodontitis [1,5]. However, the complex anatomy of the root canal system—including isthmuses, fins, lateral canals, and apical ramifications—limits the penetration and effectiveness of conventional syringe irrigation [4,44]. Activated irrigation techniques were developed to overcome these limitations. In the case of PUI, acoustic streaming and cavitation improves irrigant penetration and promotes the removal of debris and bacterial biofilm from inaccessible areas [7,45]. Similarly, laser-assisted irrigation generates photoacoustic shock waves capable of enhancing irrigant dynamics and disinfection efficacy throughout the root canal system [46,47]. Consequently, the higher healing rates observed in the present review may reflect a more effective reduction of residual microbial load, which could ultimately favor periapical healing and the subsequent repair of periradicular tissues.

### 4.3. Comparison with Previous Results

The findings of the present review are partially consistent with previous systematic reviews evaluating activated irrigation techniques in endodontics. Most earlier reviews have focused on surrogate outcomes such as bacterial reduction, smear layer removal, debris elimination, or postoperative pain rather than on the healing of apical periodontitis itself [7,45]. For example, Silva et al. (2019) [14] concluded that, although passive ultrasonic irrigation improved canal cleanliness and disinfection, the available clinical evidence was insufficient to demonstrate a clear benefit in terms of periapical healing. The results of the present meta-analysis extend those observations by suggesting that the enhanced disinfection achieved through irrigant activation may indeed translate into improved long-term healing outcomes. This discrepancy may be explained by the inclusion of more recent randomized controlled trials, particularly those incorporating advanced laser-assisted protocols such as SWEEPS and Er:YAG-based activation, together with longer follow-up periods and more rigorous radiographic assessment methods [25,47]. Furthermore, unlike previous reviews, the present study specifically focused on clinical and radiographic healing outcomes and excluded studies involving systemic antibiotic therapy, thereby minimizing an important source of confounding and allowing a more direct assessment of the contribution of intracanal disinfection to periapical healing. Consequently, the current evidence provides stronger support for a clinically relevant association between activated irrigation and the resolution of periapical lesions.

### 4.4. PUI Subgroup

With respect to passive ultrasonic irrigation, the subgroup analysis demonstrated a significantly greater probability of periapical healing compared with conventional irrigation (OR = 1.95; 95% CI: 1.08–3.54). Although the magnitude of the effect was moderate, the absence of statistical heterogeneity among studies suggests that the beneficial effect of PUI was consistent across different clinical settings and treatment protocols. These findings are in agreement with previous laboratory and microbiological investigations showing that ultrasonic activation improves irrigant penetration, biofilm disruption, and debris removal, particularly in anatomically complex regions that are inaccessible to mechanical instrumentation alone [7,45]. Interestingly, while most individual clinical trials included in the present review failed to demonstrate statistically significant differences when analyzed separately, the pooled estimate revealed a significant overall benefit. This observation highlights the value of quantitative synthesis in detecting clinically relevant effects that may remain undetected in underpowered individual studies. Therefore, although the additional benefit provided by PUI appears to be modest, the present findings support its use as an adjunctive disinfection strategy to enhance the healing potential of endodontically treated teeth with apical periodontitis.

### 4.5. Laser-Assisted Irrigation (LAI) Subgroup

The subgroup analysis of laser-assisted irrigation demonstrated a substantially greater probability of periapical healing compared with conventional irrigation (OR = 6.77; 95% CI: 1.63–28.12). Although this effect estimate was considerably larger than that observed for PUI, it should be interpreted with caution because it was derived from a limited number of studies, relatively small sample sizes, and wide confidence intervals. Consequently, the magnitude of the observed association remains uncertain and requires confirmation in future well-designed randomized clinical trials.

Although no statistical heterogeneity was detected (I^2^ = 0%), this finding should be interpreted with caution because heterogeneity statistics have limited power when only a small number of studies are included. Therefore, the absence of statistical heterogeneity does not necessarily imply complete clinical homogeneity among the included trials.

The favorable outcomes associated with laser-assisted irrigation may be explained by the unique photoacoustic phenomena generated by laser activation. In Er:YAG-based systems such as photon-induced photoacoustic streaming (PIPS) and shock-wave enhanced emission photoacoustic streaming (SWEEPS), the high absorption of laser energy by irrigant solutions generates photoacoustic shock waves that improve irrigant penetration, fluid movement, and biofilm disruption throughout the root canal system [46,47]. Other laser systems, including Er,Cr:YSGG and Nd:YAG lasers, have also been investigated for endodontic disinfection, although their mechanisms of action differ because of their distinct absorption characteristics and interactions with irrigant solutions. These technologies may contribute to bacterial reduction and enhanced cleaning of inaccessible anatomical areas, which could favor periapical healing. The present findings are consistent with previous experimental studies demonstrating superior irrigant activation and biofilm removal with laser-assisted techniques compared with conventional irrigation [46,47]. However, clinical evidence remains limited, and the available studies differ considerably in terms of laser wavelength, activation protocols, outcome definitions, and follow-up periods. Therefore, although the current results suggest a potentially greater benefit of laser-assisted irrigation than passive ultrasonic irrigation, direct comparisons between these activation methods should be interpreted cautiously. Additional randomized controlled trials with standardized protocols and long-term radiographic assessment are needed to establish the true magnitude of the clinical benefit associated with laser-assisted irrigation.

### 4.6. Influence of Systemic Antibiotics on the Interpretation of Healing Outcomes

A distinctive feature of the present review was the exclusion of studies involving systemic antibiotic therapy. This methodological decision was adopted to minimize a potential source of confounding and to allow a more direct evaluation of the relationship between intracanal disinfection and periapical healing. Although systemic antibiotics may contribute to the resolution of acute infections in selected clinical situations, their routine use in endodontic treatment is not recommended because the primary source of infection is located within the root canal system and must be managed through adequate chemo-mechanical debridement and disinfection. Consequently, the healing outcomes observed in the included studies can be more confidently attributed to the effectiveness of the local irrigation protocols rather than to adjunctive systemic antimicrobial effects. This approach is particularly relevant in light of current international guidelines, which restrict antibiotic prescription to cases presenting systemic involvement, spreading infection, or medically compromised patients at increased risk of infection-related complications [48]. Therefore, the present findings provide clinically meaningful evidence regarding the true contribution of activated irrigation techniques to periapical healing under contemporary evidence-based treatment conditions.

### 4.7. Strengths and Limitations

Several strengths of the present systematic review should be acknowledged. First, the review was conducted according to PRISMA guidelines and followed a predefined protocol, ensuring methodological transparency and reproducibility. Second, only randomized controlled trials were included, thereby maximizing the level of clinical evidence available for the research question. Third, the risk of bias and certainty of evidence were evaluated using the RoB 2 and GRADE approaches, respectively, providing a structured assessment of the methodological quality and reliability of the findings. In addition, the absence of statistical heterogeneity in both the overall and subgroup meta-analyses increased confidence in the consistency of the observed treatment effects. Finally, by specifically focusing on healing outcomes and excluding studies involving systemic antibiotic therapy, this review addressed a clinically relevant question that has received limited attention in previous evidence syntheses.

Despite these strengths, several limitations should be considered when interpreting the results. Although no statistical heterogeneity was detected (I^2^ = 0%), this finding should be interpreted with caution because heterogeneity statistics have limited power when only a small number of studies are included. Therefore, the absence of statistical heterogeneity does not necessarily imply complete clinical homogeneity among the included trials. The number of available randomized controlled trials was relatively small, and some studies included limited sample sizes, which may have reduced the precision of individual effect estimates. Considerable methodological variability was also observed regarding irrigation protocols, activation parameters, follow-up duration, definitions of treatment success, and radiographic assessment methods. While some investigations evaluated healing using conventional periapical radiography, others incorporated CBCT imaging, which is more sensitive for detecting and monitoring periapical lesions and may have influenced outcome assessment. Furthermore, one study was judged to have a high risk of bias and three studies presented some concerns according to the RoB 2 assessment, resulting in a moderate overall certainty of evidence. Finally, the limited number of studies evaluating laser-assisted irrigation restricts the strength of conclusions that can be drawn regarding the comparative effectiveness of different activation technologies. Therefore, the findings should be interpreted with appropriate caution despite the overall consistency of the pooled estimates.

### 4.8. Clinical Implications and Future Research

From a clinical perspective, the findings of the present review suggest that advanced irrigant activation techniques may be associated with improved healing outcomes, providing a meaningful adjunct to conventional chemomechanical preparation in the management of apical periodontitis. Although conventional irrigation remains the standard of care and achieved favorable healing rates in all included studies, the pooled analysis indicates that activation methods were associated with a higher probability of periapical healing. This potential benefit may be particularly relevant in teeth with complex root canal anatomy, large periapical lesions, persistent infections, or cases requiring retreatment, where achieving adequate disinfection represents a greater clinical challenge. Consequently, the incorporation of activation protocols such as PUI or LAI may contribute to more predictable treatment outcomes without the need for adjunctive systemic antibiotic therapy.

Nevertheless, the clinical applicability of these findings should be interpreted in the context of real-world practice. The included studies were conducted under controlled clinical conditions, whereas routine endodontic care is influenced by factors such as operator experience, case complexity, equipment availability, treatment costs, and adherence to standardized irrigation protocols. In particular, laser-assisted irrigation may be limited by the need for specialized equipment and additional training, whereas passive ultrasonic irrigation is more widely available and readily incorporated into general dental practice. Therefore, although the present findings support the use of activated irrigation as an adjunctive disinfection strategy, their generalizability to all clinical settings should be interpreted with caution.

Additional high-quality clinical research is required to strengthen the available evidence. Future randomized controlled trials should employ standardized irrigation protocols, uniform healing criteria, and extended follow-up periods of at least 24 months to better assess long-term periapical repair. The routine use of CBCT-based outcome assessment should also be encouraged, given its superior sensitivity for detecting periapical healing compared with conventional radiography. Furthermore, direct head-to-head comparisons between PUI and different laser-assisted irrigation systems are needed to determine whether one activation strategy provides clinically relevant advantages over another. Such studies would help establish evidence-based recommendations regarding the optimal activation protocol for improving periapical healing after root canal treatment.

## 5. Conclusions

Within the limitations of the available evidence, activated irrigation techniques were associated with significantly higher clinical and radiographic healing rates of apical periodontitis compared with conventional syringe-and-needle irrigation. Both passive ultrasonic irrigation (PUI) and laser-assisted irrigation (LAI) were associated with favorable healing outcomes, although the magnitude of the observed association varied among studies and activation methods.

The findings of this systematic review and meta-analysis suggest that enhanced intracanal disinfection may be associated with a more predictable resolution of periapical lesions without the need for adjunctive systemic antibiotic therapy. However, the available evidence is based on a limited number of randomized controlled trials and was rated as having moderate certainty. In particular, the evidence regarding laser-assisted irrigation remains limited and should be interpreted with caution. Therefore, the magnitude and clinical relevance of the observed associations require further confirmation.

Further well-designed randomized controlled trials with standardized irrigation protocols, CBCT-based outcome assessment, and longer follow-up periods are needed to confirm these findings and to determine the comparative effectiveness of different irrigation activation techniques.

## Figures and Tables

**Table 1:** Search strategies used for the identification of eligible studies in each electronic database.

PubMed	(“Periapical Diseases”[MeSH Terms] OR “apical periodontitis” OR “periapical pathology” OR “periapical infection” OR “periapical lesion” OR “periapical radiolucency”) AND (“Endodontics”[MeSH Terms] OR “Root Canal Therapy”[MeSH Terms] OR “endodontics” OR “root canal therapy” OR “root canal treatment” OR “non-surgical root canal treatment” OR “endodontic treatment”) AND (“ultrasonic irrigation” OR “passive ultrasonic irrigation” OR PUI OR “ultrasonic activation” OR “passive ultrasonic activation” OR “ultrasonically activated irrigation” OR “laser-activated irrigation” OR LAI OR “laser-assisted irrigation” OR “photon-induced photoacoustic streaming” OR PIPS OR “shock-wave-enhanced emission photoacoustic streaming” OR SWEEPS OR “diode laser activation”)
Scopus	TITLE-ABS-KEY((“apical periodontitis” OR “periapical disease*” OR “periapical lesion*” OR “periapical radiolucenc*” OR “periapical patholog*” OR “periapical infection*”) AND (“root canal treatment” OR “root canal therapy” OR endodontic* OR “non-surgical endodontic treatment”) AND (“passive ultrasonic irrigation” OR PUI OR “ultrasonic irrigation” OR “ultrasonic activation” OR “laser activated irrigation” OR “laser-assisted irrigation” OR LAI OR PIPS OR SWEEPS OR “photon-induced photoacoustic streaming” OR “shock-wave-enhanced emission photoacoustic streaming” OR “Er:YAG” OR “Er,Cr:YSGG”))
EMBASE	(‘apical periodontitis’/exp OR ‘periapical disease*’:ti,ab,kw OR ‘periapical lesion*’:ti,ab,kw OR ‘periapical radiolucenc*’:ti,ab,kw OR ‘periapical patholog*’:ti,ab,kw OR ‘periapical infection*’:ti,ab,kw) AND (‘root canal treatment’/exp OR ‘root canal therapy’:ti,ab,kw OR ‘root canal treatment’:ti,ab,kw OR endodontic*:ti,ab,kw OR ‘non-surgical endodontic treatment’:ti,ab,kw) AND (‘passive ultrasonic irrigation’:ti,ab,kw OR pui:ti,ab,kw OR ‘ultrasonic irrigation’:ti,ab,kw OR ‘ultrasonic activation’:ti,ab,kw OR ‘laser activated irrigation’:ti,ab,kw OR ‘laser-assisted irrigation’:ti,ab,kw OR lai:ti,ab,kw OR pips:ti,ab,kw OR sweeps:ti,ab,kw OR ‘photon-induced photoacoustic streaming’:ti,ab,kw OR ‘shock-wave-enhanced emission photoacoustic streaming’:ti,ab,kw OR ‘er:yag’:ti,ab,kw OR ‘er,cr:ysgg’:ti,ab,kw)
WoS	TS=((“apical periodontitis” OR “periapical disease*” OR “periapical lesion*” OR “periapical radiolucenc*” OR “periapical patholog*” OR “periapical infection*”) AND (“root canal treatment” OR “root canal therapy” OR endodontic* OR “non-surgical endodontic treatment”) AND (“passive ultrasonic irrigation” OR PUI OR “ultrasonic irrigation” OR “ultrasonic activation” OR “laser activated irrigation” OR “laser-assisted irrigation” OR LAI OR PIPS OR SWEEPS OR “photon-induced photoacoustic streaming” OR “shock-wave-enhanced emission photoacoustic streaming” OR “Er:YAG” OR “Er,Cr:YSGG”))

**Table 2:** Excluded studies and their reasons for exclusion.

Reasons for Exclusion	Excluded Studies
Absence of control group (*n* = 8)	Almeida et al., 2023 [27] Pazin et al., 2024 [28] Ahmedbeyli et al. 2020 [29] Pantaleo et al., 2022 [30] Parashar, 2024 [31] Herrera et al., 2017 [32] Wan et al., 2017 [33] Ustun et al., 2026 [34]
Use of sonic irrigation (*n* = 3)	Arikan et al., 2024 [35] Montero et al., 2025 [36] Kolberg-Babrzyńska et al., 2025 [37]
Follow-up shorter than 6 months (*n* = 4)	Zhao et al., 2024 [38] Middha et al., 2017 [39] Montaser et al., 2023 [40] Dagher et al., 2019 [41]
Ineligible patient population (*n* = 1)	Asli Usta Tahmaz, 2026 [42]
Non-longitudinal study desing (*n* = 2)	Meire et al., 2022 [13] Orozco et al., 2020 [43]

**Table 3:** Characteristics of included studies.

Author/Year/Country	Type of Study	Sample (n)	Radiographic Evaluative	Population	Diagnostic	PUI/LAI	Control Group	Success Rate (%)	Conclusions
Verma et al. 2020 [22] India	Randomized control trial (RCT)	69 teeth in 3 groups (n = 23 per group: G1, G2 y G3)	Periapical radiographs and CBCT	69 patients 18–45 years old	Necrosis and chronic apical periodontitis (CAP) PAI index between 3 and 5	NaOCl 3%, 2 mL Activation 20 s in 4 cycles PUI and LAI	NaOCl 3% 2 mL	6M: *p* = 0.18; G1 (94.4%); G2 (83.3%); G3 (72.2%) 12M: *p* = 0.05 overall; G1 (26.3%); G2 (47.4%); G3 (42.1%)	Overall success rate: PUI 100%; LAI 100%; control 78.9% (*p* = 0.014)
Tang et al. [23] China	RCT	300 teeth in 3 groups (n = 100)	Periapical radiographs	300 patients 60–72 years old	CAP	Only PUI NaOCL 2.5% vs. active silver ion antibacterial solution	NaOCl 2.5%	6M: *p* = 0.375; G1 (80.83%); G2 (81.15%); G3 (74.58%) 12M: *p* = 0.078; G185%); G2 (88.52%); G3 (77.97%)	Success rates were higher in the groups with PUI, although without statistically significant differences between groups at 6 and 12 months.
Liang et al. 2013 [24] China	RCT	84 teeth (PUI group n = 40; control group n = 44)	Periapical radiographs (PR) and CBCT	84 patients 18–69 years old	CAP	Only PUI NaOCl 5.25% Activation 10 s in 3 cycles	NaOCl 5.25%	Teeth without radiolucency in CBCT (19%) vs. PR (32.1%) (*p* = 0.038) Lesion volume (CBCT): initial volume of 1.5–375.4 mm^3^, which was reduced by 80–100% in 54/84 teeth (64%) during follow-up (10–19 months).	Overall success rate PUI 95.1% vs. control 88.4% (*p* = 0.470)
Dogan et al. 2024 [25] Turkey	RCT	56 teeth	Periapical radiographs PAI index ≥ 3	56 patients 18–65 years old	CAP	Group 1: NaOCl 2.5% MDA 6 mL 1 min Group 2: PUI + NaOCl 2.5% 2 mL 20 s in 3 cycles Group 3: SWEEPS laser Er:YAG activation NaOCl 2.5% 2 mL 3 × 20 s	NaOCl 2.5% 6 mL	Significant lesion reduction pre- and post-treatment (*p* < 0.05). Totally healed: 20%; reduced lesion: 67%; unchanged: 11%; no cases of lesion enlargement. Intergroup comparison of the % reduction: Kruskal–Wallis = 2.855 (*p* = 0.414)	Overall success rate SWEEPS 86.9%; PUI 85.4%; MDA 80.4%; control 74.5%
Martins et al. 2014 [26] Portugal	RCT	43 teeth 30 of them 12 months follow up (control n = 12; LAI n = 18)	--	43 adult patients	Necrosis and CAP	LAI Er,Cr:YSGG (2780 nm) with radial-emitting tips, irrigation in 2 appointments (4 cycles per appointment)	NaOCl 3% 5 mL	Significant decrease in PAI at 12 months (*p* < 0.05). No statistical difference in PAI at 12 months (*p* > 0.05). Healing (PAI ≤ 2): control 100% vs. laser 88.9% → no statistical difference was found (*p* > 0.05)	Er,Cr:YSGG laser-assisted endodontic treatment is at least equally effective as the conventional sodium hypochlorite and calcium hydroxide protocol, demonstrating comparable clinical and radiographic periapical healing at 12 months.

**Table 4:** Intergroup comparison of treatment success rates.

Author/Year/Country	Sample (n)	PUI		Laser		Control		Odds Ratio (OR)
		No. (%)	%	No. (%)	%	No. (%)	%	
Verma et al., 2020 India [22]	57	19 (19)	100.00	19 (19)	100.00	19 (15)	78.9	PUI OR = 11.32 Laser OR = 11.32
Tang et al., 2015 China [23]	238	120 (102)	85	-	-	118 (92)	77.97	OR = 1.60
Liang et al., 2013 China [24]	84	41 (39)	95.12	-	-	43 (38)	88.37	OR = 2.56
Doğan et al., 2024 Turkey [25]	42	14 (14)	100.00	14 (13)	92.85	14 (11)	78.57	PUI OR = 8.83 Laser OR = 3.55
Martins et al., 2014 Portugal [26]	30	-	-	18 (18)	100.00	12 (10)	83.33	OR = 8.82
Total	451	194 (174)	89.69	51 (50)	98.03	206 (166)	80.58	OR = 2.25

Treatment success rates correspond to the criteria reported by each original study and were used for the quantitative synthesis. These values should not be directly compared with individual radiographic outcome categories reported in Table 3.

**Table 5:** GRADE assessment of evidence certainly.

Certainty Assessment						Certainty Assessment	Importance
Nº Studies	Study Design	Risk of Bias	Inconsistency	Indirectness	Imprecision		
5	RCT	Serious ^a^	Not serious ^b^	Not serious	Not serious	Moderate **(⊕⊕⊕◯)**	Critical/Important

Footnotes: ^a^ Downgraded one level due to concerns regarding risk of bias, as three studies were judged as having some concerns and one study was classified as high risk of bias. ^b^ No statistical heterogeneity was detected among studies; I^2^ = 0; Q test for heterogeneity: *p* = 0.557.

## Data Availability

All data generated or analyzed during this study are included in this published article (and its Supplementary Materials files).

## Supplementary Materials

The following supporting information can be downloaded at: https://www.mdpi.com/article/10.3390/jcm15145397/s1, Table S1: PRISMA 2020 Checklist.
